# Updating Disyllabic Word Lists for Italian Speech Audiometry

**DOI:** 10.1002/lio2.70572

**Published:** 2026-09-29

**Authors:** Laura Biondi, Marco Pozzi, Alessandro Martini, Federica Di Berardino

**Affiliations:** ^1^ Dipartimento di Studi Letterari, Filologici e Linguistici University of Milan Milan Italy; ^2^ Audiology Unit IRCCS Fondazione Ca' Granda Ospedale Maggiore Policlinico Milan Italy; ^3^ Health Professions Management, IRCCS Foundation Ca' Granda Ospedale Maggiore Policlinico Milan Italy; ^4^ Honorary Professor of Otorhinolaryngology, Department of Neurosciences University of Padua Padua Italy; ^5^ Section of Otorhinolaryngology‐Head and Neck Surgery, Department of Neurosciences University of Padua Padua Italy; ^6^ Department of Clinical Sciences and Community Health, Dipartimento di Eccellenza 2023‐2027 University of Milan Milan Italy

**Keywords:** bisyllabic, disyllabic, Italian material, speech audiometry, speech test, word

## Abstract

**Objective:**

To describe a standardized clinical for Italian speech audiometry using new disyllabic word lists and to report preliminary validation and normative parameters in normal‐hearing subjects.

**Methods:**

In accordance with ISO 8253‐3 guidelines, phonetically balanced disyllabic word lists were administered to 12 normal‐hearing native Italian speakers (aged 18–25 years, pure‐tone average ≤ 10 dB HL). Word Recognition Scores (WRS) were assessed across a descending intensity gradient (30–0 dB HL) to derive psychometric functions. Inter‐list equivalence and 1‐week test–retest reliability were evaluated in quiet and background noise (cocktail party noise). Reliability was quantified using intraclass correlation coefficients (ICC), Bland–Altman analysis, and repeated‐measures ANOVA.

**Results:**

Mean speech recognition threshold (SRT50%) was 5.9 ± 2.2 dB HL, with a steep, highly sensitive average slope of 4.9%/dB near threshold. Maximum intelligibility (SRT 100%) was reached at 30 dB HL, across all lists. Intrasubject test‐list variability was negligible (< 5% difference, ANOVA *p* > 0.05). Bland–Altman showed high temporal stability (mean bias: +0.88%; Limits of Agreement: ±1.5 words/list). Test–retest reliability was excellent (ICC = 0.957, 95% CI: 0.932–0.973, *p* < 0.001). In background noise, stable reproducibility was maintained, showing a well‐contained coefficient of variation (14.5%–14.7%) at SNR 0 dB and a perfect ceiling effect at SNR +10. Good equivalence among lists were observed.

**Conclusion:**

The new 10‐item lists demonstrate robust equivalence and high test–retest reliability under ISO criteria. This validation workflow offers a time‐efficient paradigm that is methodologically applicable to other languages, supporting standardized cross‐linguistic clinical assessments.

**Level of Evidence:**

4.

## Graphical abstract

While speech audiometry materials worldwide frequently suffer from outdated language use, modern technology allows for linguistic optimization. This study establishes a universal framework to update speech materials by balancing lists according to contemporary web‐derived text corpora and word frequency usage, successfully demonstrated through the development of the Italian Bocca 2.0 lists.

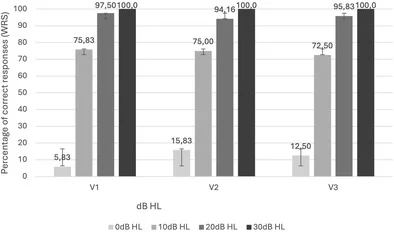

## 1 Introduction

Standard Italian speech audiometry traditionally uses disyllabic word lists by Bocca and Pellegrini in the 1950s [1], which remain the clinical gold standard to define the speech intelligibility curve is no more representative of the actual used language. In 1993, Turrini et al. [2] proposed new materials to better reflect contemporary language use and ensure more rigorous phonemic and lexical balance across the corpus; however, increasing the list length (from 10 to 20 words) caused users to different apply this material and more frequently to randomly select 10‐word subsets; this inconsistent application completely eliminated in clinical practice the phonological balance among the lists. Additionally, variability in recording levels and calibration tones in the different published materials provides a further critical limitation [3]. While the Matrix Sentence Test has been widely adopted in multiple languages, including Italian [4], it complements rather than replaces traditional speech audiometry, that remains essential for deriving psychometric functions, thus highlighting the need for updating the old standardized speech material of 1950s. Differently from Turrini et al. [2], the new proposed verbal material of this study was limited to protonyms and avoids the use of verbs and adverbs or pronouns (Table 1). Additionally to the previous disyllabic versions, a computational analysis of the actual usage frequency in different corpora (written and web‐based) was introduced to refine the word selection.

**TABLE 1:** Word lists [Bocca 2.0].

Lista 1	LISTA 2	LISTA 3	LISTA 4	LISTA 5	LISTA 6	LISTA 7	LISTA 8	LISTA 9	LISTA 10
Bocca	Latte	Gioia	Piante	Tende	Maggio	Braccio	Pasta	Calma	Ansia
Mondi	Corpi	Pranzo	Giorni	Giacca	Crisi	Spesa	Occhio	Mostra	Brano
Lago	Dente	Borse	Quote	Nodi	Vetro	Giochi	Classe	Gesti	Pesce
Olio	Faccia	Via	Draghi	Metro	Scale	Scene	Fiume	Gruppi	Fuochi
Testi	Vino	Sole	Caccia	Lingua	Prove	Tetto	Svolta	Calcio	Volta
Fine	Guide	Sguardi	Ville	Pietre	Fiore	Fila	Gradi	Squadre	Tempo
Centro	Schermo	Fumo	Figlio	Volti	Nido	Viso	Spiagge	Voli	Specie
Stampe	Strisce	Carni	Buco	Gusto	Gare	Luogo	Bordo	Base	Modi
Chiavi	Giugno	Sedia	Norma	Libri	Dubbi	Mezzi	Scienze	Forze	Grazie
Euro	Bacio	Fronte	Senso	Sforzo	Cielo	Studio	Ladri	Monti	Pugni

LISTA 11	LISTA 12	LISTA 13	LISTA 14	LISTA 15	LISTA 16	LISTA 17	LISTA 18	LISTA 19	LISTA 20
Leggi	Cibo	Cerchio	Cane	Moglie	Bosco	Frutto	Onde	Mamma	Tipi
Fuga	Radio	Sangue	Roba	Fianco	Cura	Donna	Sera	Uva	Strega
Tracce	Prezzi	Nonna	Piazze	Luce	Fondo	Specchi	Cambio	Carte	Luna
Madri	Stelle	Membro	Dati	Droga	Serie	Guscio	Frase	Ritmo	Soldi
Posta	Uomo	Voglia	Ombre	Scelta	Campi	Teste	Uovo	Gola	Fascia
Fede	Versi	Danni	Genio	Pelle	Gloria	Mente	Guardia	Piedi	Banche
Sposi	Punte	Festa	Flotta	Vista	Voti	Spazio	Giri	Scuola	Diete
Viaggio	Forma	Muro	Scuse	Trono	Cifra	Vento	Letto	Gente	Scopo
Barca	Colpe	Raggi	Gatti	Suoni	Liste	Labbra	Padre	Fretta	Marzo
Treno	Gamba	Spalle	Nave	Erba	Zona	Chiodi	Luglio	Beni	Vita

The aim of this study was to introduce an update of disyllabic word lists for Italian speech audiometry and, in accordance with ISO standards, to report the results of a validation prospective observational methodological study in normal‐hearing subjects including test–retest reliability, inter‐list variability, and its relative performance compared with traditional old materials for their clinical implementation.

## 2 Methods

### 2.1 Participants

In accordance with ISO 8253‐3 standards for the validation of speech materials (20 lists of 10 word each), a group of 12 normal‐hearing native Italian speakers, healthy volunteers (young adults, aged 18–25 years) was recruited. Inclusion criteria were strictly defined by a pure‐tone average (PTA) ≤ 10 dB HL across the frequency range of 0.125–8 kHz and no history of otological disease, middle ear surgery, or vestibular dysfunction and of neuropsychological or neurodevelopmental disorders, such as specific learning disabilities, to eliminate potential confounding factors in speech perception. All participants were volunteers, provided informed consent and anonymity has been guaranteed.

### 2.2 Speech Material

Phonetically balanced disyllabic word lists were developed to ensure comparable phonetic distribution, lexical familiarity and frequency of use among lists (Table 1). Recordings were produced by a professional native speaker in a sound‐treated environment and were normalized for intensity.

### 2.3 Procedure

To verify phonological balance of lists, inter‐list equivalence, and slope of intelligibility functions using 10 dB HL step increments, speech recognition scores were measured through headphones (Radioear 3045) by an audiometer (Inventis Harp Plus) at fixed intensities from 30 to 0 dB HL in the same day twice after a break of 1 h: the first time (V1) word lists were presented according to their original recording order, assigned sequentially across participants to ensure balanced use of all lists and the second time (V2) using a randomized allocation of word lists. To minimize recall effects, no list in V2 was presented at the same intensity level as in V1, and each list was administered at least twice within the same day at different presentation levels (Table 2). One week later (V3), the same participants underwent a third evaluation (V3) using the same testing procedure adopted at V1 in order to assess test–retest reliability and the reproducibility of the psychometric function. Both inter‐list equivalence (V1–V2) and test–retest reliability (V1–V3) were assessed. Within the same sections and in the same conditions, the traditional speech test with Bocca and Pellegrini materials has also been tested (C1 with V1) and C2 1 week‐ later together with V3.

**TABLE 2:** Procedure of presenting lists.

# ID	Test	Quiet		
		V1 test	V2 (random) retest	V3 retest
		Lists	Lists	Lists
1	30	1	7	1
	20	2	5	2
	10	3	8	3
	0	4	6	4
2	30	1	6	1
	20	2	8	2
	10	3	5	3
	0	4	7	4
3	30	3	11	3
	20	4	9	4
	10	5	12	5
	0	6	10	6
4	30	5	10	5
	20	6	12	6
	10	7	9	7
	0	8	11	8
5	30	6	15	6
	20	7	13	7
	10	8	16	8
	0	9	14	9
6	30	9	14	9
	20	10	16	10
	10	11	13	11
	0	12	15	12
7	30	10	19	10
	20	11	17	11
	10	12	20	12
	0	13	18	13
8	30	11	18	11
	20	12	20	12
	10	13	17	13
	0	14	19	14
9	30	13	3	13
	20	14	1	14
	10	15	4	15
	0	16	2	16
10	30	15	2	15
	20	16	4	16
	10	17	1	17
	0	18	3	18
11	30	17	2	17
	20	18	7	18
	10	19	12	19
	0	20	17	20
12	30	17	4	17
	20	18	9	18
	10	19	14	19
	0	20	19	20

Thereafter, in accordance with ISO 8253‐3 standards, 1 week after, WRS have also been tested in background noise (cocktail party noise), having speech material delivered through loudspeaker by a Aurical2 (Natus Medical, Otometrics). The reproducibility in noise was tested at a signal‐to‐noise ratio (SNR) of 0 (60/60 dB SPL) and +10 (60/50 dB SPL) with a test–retest of both condition using randomly selected lists, within the same day. Testing was all performed in a sound‐attenuated booth room, and the system was calibrated using a Class 1 sound level meter in accordance with IEC 61672‐1 standards.

### 2.4 Statistical Analysis

Statistical analyses were performed using SPSS software (release 24.0; IBM Corp., Armonk, NY, USA). Descriptive statistics were computed for all the variables. Additionally, speech detection threshold (SDT), speech recognition threshold (L50), maximum intelligibility threshold (L100), clarifying the rate of increase in intelligibility per dB, were mathematically derived. Wilcoxon signed‐rank tests, Bland–Altman analysis, Cronbach's alpha, and ICC evaluated inter‐list equivalence and 1‐week test–retest reliability, while repeated‐measures and one‐way ANOVA assessed intensity effects and inter‐list variability, with significance set at *p* < 0.05.

### 2.5 Ethical Committee Approval

This prospective observational study was conducted in strict observance of the World Medical Association's Declaration of Helsinki and garnered the ethical approval from the Institutional Review Board of the University of Milan, Italy (IRB 20.26, March 17, 2026). Written informed consent for participation and publishing of the results was obtained from all participants. Anonymity has been guaranteed.

## 3 Results

The mean age was 22.0 ± 0.8 (six females; six males), PTA 10.0 dB HL ±0.0 dB HL.

### 3.1 Perceptual Equivalence and Intensity‐Response Functions

The percentage of correct answers obtained across a descending intensity gradient (30, 20, 10, and 0 dB HL) at basal condition (V1), with randomly proposed lists (V2) and at after 1‐week retest of V1 are reported in Figure 1. The differences among testing conditions were < 5%. In the nonparametric Wilcoxon Signed Ranks test for dependent groups, the comparison did not show a significant statistical difference, except for SDT for V1/V2/V3 and C1/2 at speech discrimination score (SDS) of 0%. The coefficient of variation was very low at all the intensities. Raw data are reported in Table 3.

**FIGURE 1:**
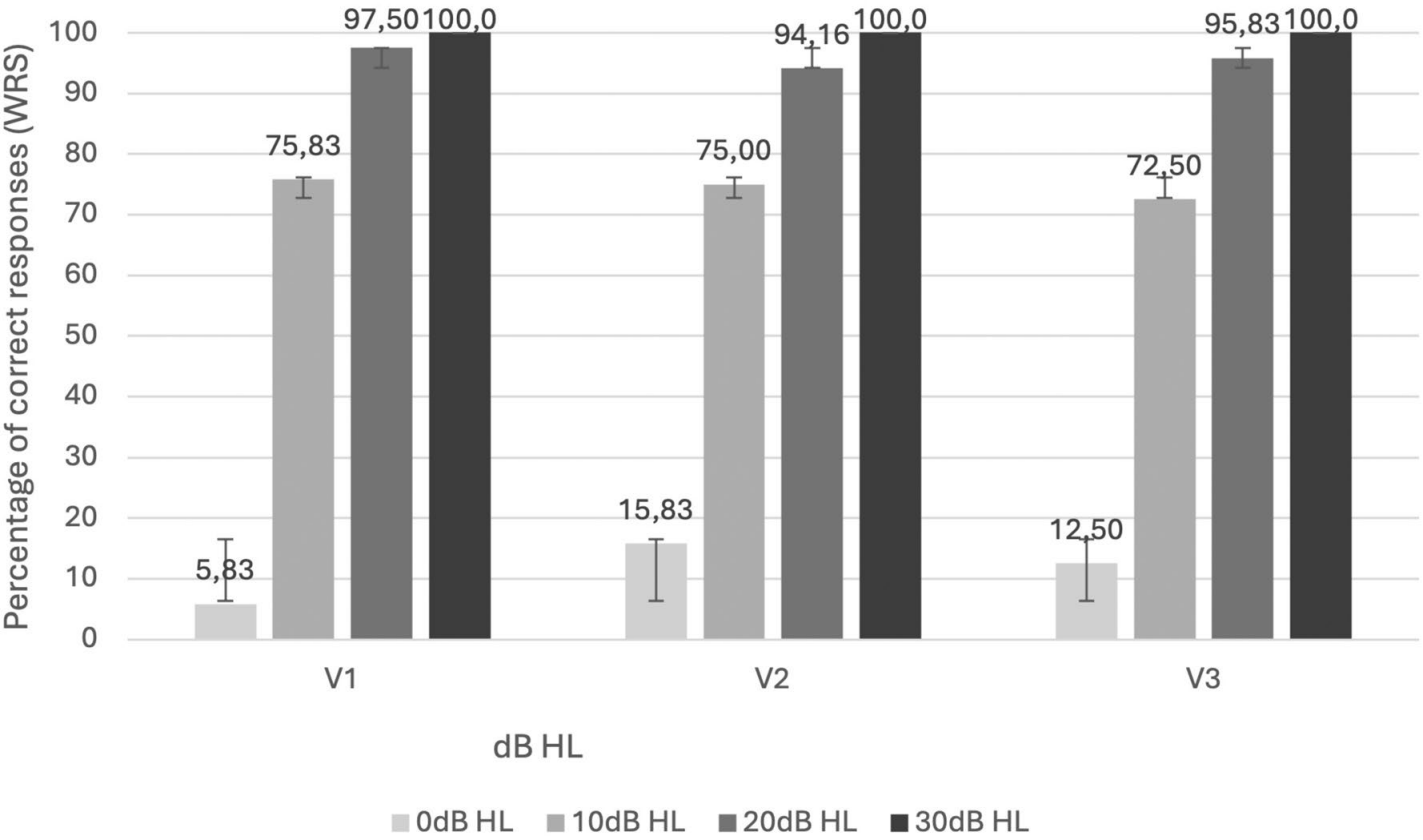
The slope of the psychometric function.

**TABLE 3:** Raw data.

QUIET condition (presentation levels)	Test (V1)	Retest (V2_ random)	Retest (V3_one‐week after)	Traditional speech test (C1)	Traditional speech test retest (C2)
30 dB HL	100% ± 0%	100% ± 0%	100% ± 0%	100% ± 0%	100% ± 0%
20 dB HL	97% ± 6%	94% ± 5%	96% ± 8%	97% ± 5%	98% ± 6%
10 dB HL	75% ± 16%	75% ± 12%	72% ± 20%	78% ± 14%	67% ± 18%
0 dB HL	6% ± 9%	16% ± 12%	12% ± 15%	23% ± 14%	24% ± 21%
SDT	−1% ± 1%	−3% ± 3%	−2% ± 3%	−4% ± 3%	−12% ± 18%
L50	7% ± 2%	6% ± 2%	7% ± 3%	5% ± 2%	5% ± 4%
L100	21% ± 5%	25% ± 7%	22% ± 6%	22% ± 7%	22% ± 4%

NOISE condition (presentation levels)	Test (V4)	Retest (V4)	Cronbach's alpha	Intra‐class Correlation (ICC)	
SNR10 (60/50 dB SPL)	100% ± 0%	100% ± 0%	No variance	No variance	
SNR0 (60/60 dB SPL)	81% ± 12%	78% ± 11%	0.812	0.815	

*Note:* The percentage of correct responses in V1, V2, V3, C1, and C2 are expressed as: mean ± standard deviation (SD). Values of ICC, ≥ 0.75 perfect reliability; 0.6–0.74, good; 0.4–0.59, medium; < 0.4, poor reliability.

### 3.2 Test–Retest Reliability and Temporal Stability

Cronbach's alpha values and Intra‐class Correlation (ICC) are reported in Table 4. ICC 0.957 (95% confidence interval: 0.932–0.973, *p* < 0.001) demonstrating excellent reproducibility. Bland–Altman analysis revealed a negligible systematic mean bias of only +0.88%, ruling out any clinically relevant learning effect. The 95% Limits of Agreement spanned from −12.5% to +14.2%, confirming that individual test–retest variability is tightly restricted within acceptable clinical tolerances (±1.5 words per list). The test with 20 dB HL stimulus showed the highest consistency (Cronbach's alpha 0.919). A repeated‐measures ANOVA confirmed a highly significant main effect of stimulus intensity on speech discrimination performance [*F* = 214.7, *p* < 0.001]. Pairwise post hoc comparisons with Bonferroni correction demonstrated significant discrimination shifts across all 10‐dB steps (*p* < 0.001). Interpolation of the cumulative intensity‐response function established the cohort's mean speech recognition threshold (SRT) at 5.9 dB HL, with a steep, highly sensitive average slope of 4.9%/dB near the 50% threshold.

**TABLE 4:** Test–retest reliability.

Comparisons	Cronbach's alpha (*α*)	Intra‐class correlation (ICC) (single‐average)
V1_0–V3_0	0.718	0.496–0.663
V1_10–V3_10	0.413	0.272–0.428
V1_20–V3_20	0.919	0.838–0.912
V1_30–V3_30	1	1
V1_0%–V3_0%	0.718	0.496–0.663
V1_50%–V3_50%	0.244	0.150–0.260
V1_100%–V3_100%	0.715	0.571–0.727
V1_0–V2_0	0.628	0.458–0.628
V1_10–V2_10	0.795	0.66–0.795
V1_20–V2_20	0.517	0.349–0.517
V1_30–V2_30	1	1
V1_0%–V2_0%	0.605	0.434–0.605
V1_50%–V2_50%	0.843	0.729–0.843
V1_100%–V2_100%	0.224	0.126–0.224
V2_0–V3_0	0.572	0.401–0.572
V2_10–V3_10	0.393	0.245–0.393
V2_20–V3_20	0.361	0.22–0.361
V2_30–V3_30	1	1
V2_0%–V3_0%	0.623	0.452–0.623
V2_50%–V3_50%	0.01	0.005–0.01
V2_100%–V3_100%	0.632	0.462–0.632

Abbreviations: V1, test; V2, random V1; V3, retest of V1.

### 3.3 Speech‐in‐Noise

At SNR +10 dB, the material yielded a perfect ceiling effect with a mean recognition score of SNR + 10 across both the initial session and the 1‐week retest (Table 3). The coefficient of variation at SNR 0 dB SPL è 14.5%–14.7% and 0% al SNR + 10.

### 3.4 Inter‐List Equivalence

Inter‐list WRS in quiet and noise are reported in Table 5. None of the lists proved to be systematically easier or more difficult across either the noise or quiet conditions and between quiet and noise conditions and across all tested intensities. In our global data, the maximum difference between list means ranged from 0% at highly stable intensities (such as 30 dB HL and +10 dB SNR) up to approximately 30%–35% at the critical 10 dB HL condition. Since these fluctuations remain purely stochastic and nonsignificant, the material is fully homogeneous and compliant with ISO 8253‐3 standards.

**TABLE 5:** Speech recognition scores across the 20 word lists in quiet and noise conditions.

	0 dB HL	10 dB HL	20 dB HL	30 dB HL	0 SNR (60/60)	+10 SNR (60/50)
List 1	10.0 ± 10.0	65.0 ± 5.8	90.0 ± 14.1	100.0 ± 0	66.7 ± 5.8	100.0 ± 0
List 2	13.3 ± 5.8	70.0 ± 14.1	92.5 ± 9.6	100.0 ± 0	83.3 ± 5.8	100.0 ± 0
List 3	10.0 ± 10.0	74.0 ± 24.1	92.5 ± 9.7	100.0 ± 0	66.7 ± 5.8	100.0 ± 0
List 4	10.0 ± 17.3	60.0 ± 14.1	92.5 ± 15.0	100.0 ± 0	66.7 ± 5.8	100.0 ± 0
List 5	6.7 ± 5.8	76.0 ± 23.0	85.0 ± 12.9	100.0 ± 0	56.7 ± 5.8	100.0 ± 0
List 6	3.33 ± 5.8	60.0 ± 14.1	92.5 ± 5.0	100.0 ± 0	66.7 ± 15.3	100.0 ± 0
List 7	20.0 ± 10.0	52.5 ± 9.6	95.0 ± 5.8	100.0 ± 0	83.3 ± 5.8	100.0 ± 0
List 8	0.0 ± 0.0	57.5 ± 15.0	87.5 ± 18.9	100.0 ± 0	60.0 ± 0.00	100.0 ± 0
List 9	3.4 ± 5.8	63.3 ± 11.5	92.5 ± 5.8	100.0 ± 0	63.3 ± 5.8	100.0 ± 0
List 10	10.0 ± 10.0	53.3 ± 11.5	75.0 ± 5.8	100.0 ± 0	76.7 ± 11.5	96.7 ± 5.7
List 11	3.4 ± 5.8	47.5 ± 9.6	95.0 ± 5.8	100.0 ± 0	70.0 ± 0	100.0 ± 0
List 12	0.0 ± 0.0	78.0 ± 13.0	85.0 ± 5.8	100.0 ± 0	63.3 ± 11.5	100.0 ± 0
List 13	0.0 ± 0.0	50.0 ± 7.1	82.5 ± 20.6	100.0 ± 0	83.3 ± 5.8	100.0 ± 0
List 14	0.0 ± 0.0	63.3 ± 15.3	85.0 ± 17.3	100.0 ± 0	60.0 ± 0.0	100.0 ± 0
List 15	6.7 ± 5.8	82.5 ± 9.6	86.7 ± 11.5	100.0 ± 0	70.0 ± 10.0	96.7 ± 5.7
List 16	6.7 ± 11.5	70.0 ± 8.2	90.0 ± 8.2	100.0 ± 0	53.3 ± 11.5	100.0 ± 0
List 17	13.3 ± 15.3	58.0 ± 11.0	85.0 ± 17.3	100.0 ± 0	73.3 ± 11.5	100.0 ± 0
List 18	6.7 ± 11.5	60.0 ± 11.5	87.5 ± 15.0	100.0 ± 0	60.0 ± 10.0	100.0 ± 0
List 19	13.3 ± 15.3	68.0 ± 19.2	86.7 ± 15.3	100.0 ± 0	70.0 ± 0.0	100.0 ± 0
List 20	6.7 ± 5.7	60.0 ± 16.3	85.0 ± 12.9	100.0 ± 0	66.7 ± 5.7	100.0 ± 0
Maximum inter‐list difference	20.0	35.0	20.0	0.0	30.0	3.3
*p*	*p* > 0.05	*p* > 0.05	*p* > 0.05	*p* > 0.05	*p* > 0.05	*p* > 0.05

*Note:* Word recognition scores (WRS), expressed as the percentage of correctly identified words, are reported as mean ± standard deviation. Repeated‐measures and one‐way ANOVA among the 20 lists (*p*‐values) did not show statistically significant differences.

As shown in Figure 2, in quiet WRS approached ceiling at 30 dB HL, with all lists achieving 100%. No statistically significant differences among the 20 word lists were observed at any presentation level, as assessed by ANOVA analysis (*p* = ns). As expected, greater variability was observed at lower presentation levels, particularly at 10 dB HL which is the steepest section of the psychometric curve. These fluctuations among the 20 lists were not consistently repeated by the same lists at higher or lower intensities.

**FIGURE 2:**
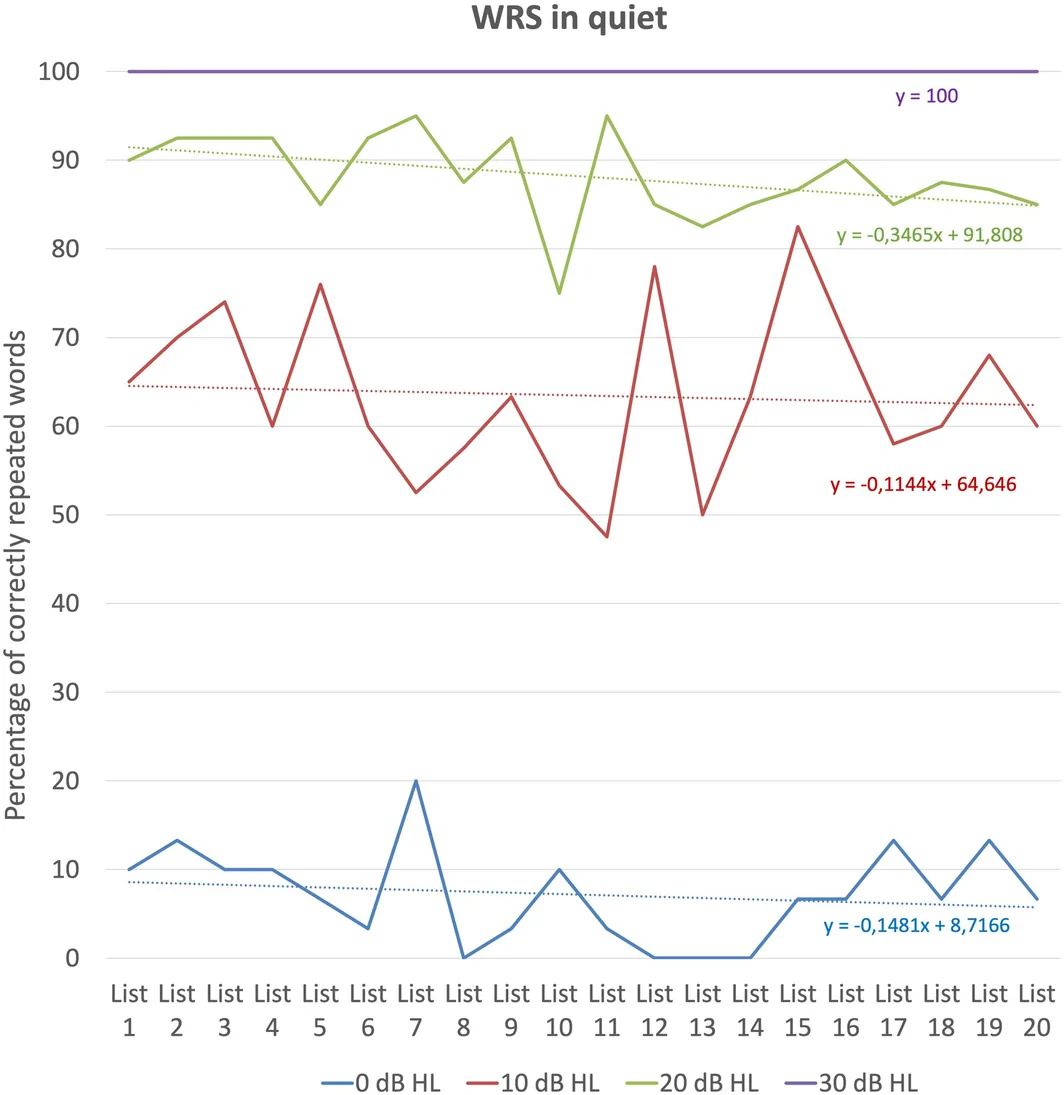
WRS in quiet condition among the 20 lists.

Figure 3 shows the results of the 20 lists in the two noise conditions. Inter‐list variability in noise was minimal at +10 dB SNR, with all lists achieving nearly 100% WRS. The ranges of mean WRS values did not overlap between the two SNR conditions, with consistently higher scores at +10 dB SNR. A higher, yet limited, variability was observed at 0 dB SNR; however, no statistically significant differences among the 20 word lists were observed at either SNR condition. indicating the absence of a systematic effect of word‐list difficulty.

**FIGURE 3:**
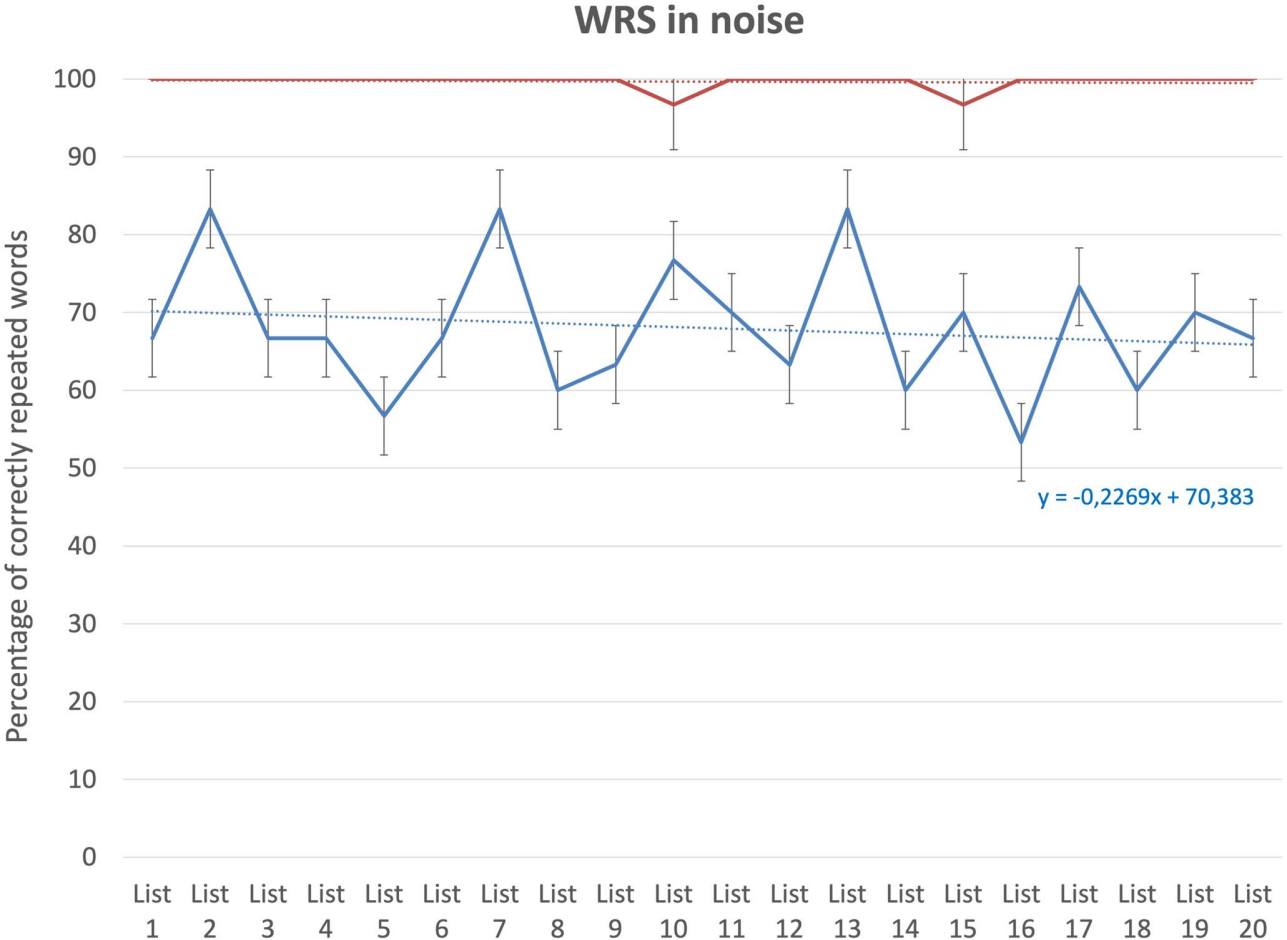
WRS in noise condition among the 20 lists.

## 4 Discussion

Speech recognition performance showed limited variability across lists. Test–retest analysis demonstrated high reliability. The material generated a highly consistent, classic sigmoidal psychometric curve with descending pattern between 20 and 0 dB HL, reaching a plateau at 20 dB HL, as expected. The comparison of the mean of the percentage of correct words between V2 and V1/V3 provides minimal differences (< 5%), confirming the minimum variability among lists. Therefore, the lists are statistically equivalent in terms of difficulty and phonetic balance. The comparison with the traditional lists (C1/C2) shows a slight phonetic familiarity effect at low intensities (0 and 10 dB), but it is not significant above 20 dB, which reduces the possibility of comparison that is already hindered by the well‐known variability of the recordings [3]. In the new lists, the absence of differences between V1 and V3 confirms that there is no learning effects after 1 week. A good level of consistency across the word lists was observed, with no statistically significant differences in WRS among lists at any presentation level. As expected, greater variability was observed at lower presentation levels, where speech recognition performance is more sensitive to individual differences in normal hearing auditory performances, rather than showing evidence of a systematic difference in word‐list difficulty. This limited variation, in fact, only occurs within the steepest portion of the speech slope function and is not consistently repeated among the same lists at the other presented intensities. Given that the statistical analysis did not reach significant differences among the lists in any of the tested conditions, these materials agree the requirements for clinical and research applications in accordance with ISO 8253‐3 guidelines. The proposed protocol provides a standardized approach to Italian speech audiometry with high reliability and limited inter‐list variability. The procedure may improve clinical consistency and facilitate comparison across centers.

## Funding

The authors have nothing to report.

## Conflicts of Interest

The authors declare no conflicts of interest.

## Data Availability

The data that support the findings of this study are openly available in Dataverse Unimi at https://doi.org/10.13130/RD_UNIMI/FMQLSZ, reference number 10.13130.
